# Starvation Promotes Autophagy-Associated Maturation of the Ovary in the Giant Freshwater Prawn, *Macrobrachium rosenbergii*

**DOI:** 10.3389/fphys.2017.00300

**Published:** 2017-05-12

**Authors:** Wilairat Kankuan, Chaitip Wanichanon, Rossella Titone, Attakorn Engsusophon, Chanudporn Sumpownon, Worawit Suphamungmee, Federica Morani, Matilde Masini, Michela Novelli, Ciro Isidoro, Prasert Sobhon

**Affiliations:** ^1^Department of Anatomy, Faculty of Science, Mahidol UniversityBangkok, Thailand; ^2^Laboratory of Molecular Pathology, Department of Health Sciences, Università del Piemonte Orientale “Amedeo Avogadro”Novara, Italy; ^3^Department of Translational Research and New Technologies in Medicine and Surgery, University of PisaPisa, Italy; ^4^Faculty of Allied Health Sciences, Burapha UniversityChonburi, Thailand

**Keywords:** *Macrobrachium rosenbergii*, starvation, autophagy, reproduction, ovarian maturation

## Abstract

Limitation of food availability (starvation) is known to influence the reproductive ability of animals. Autophagy is a lysosomal driven degradation process that protects the cell under metabolic stress conditions, such as during nutrient shortage. Whether, and how starvation-induced autophagy impacts on the maturation and function of reproductive organs in animals are still open questions. In this study, we have investigated the effects of starvation on histological and cellular changes that may be associated with autophagy in the ovary of the giant freshwater prawn, *Macrobachium rosenbergii*. To this end, the female prawns were daily fed (controls) or unfed (starvation condition) for up to 12 days, and the ovary tissue was analyzed at different time-points. Starvation triggered ovarian maturation, and concomitantly increased the expression of autophagy markers in vitellogenic oocytes. The immunoreactivities for autophagy markers, including Beclin1, LC3-II, and Lamp1, were enhanced in the late oocytes within the mature ovaries, especially at the vitellogenic stages. These markers co-localized with vitellin in the yolk granules within the oocytes, suggesting that autophagy induced by starvation could drive vitellin utilization, thus promoting ovarian maturation.

## Introduction

Autophagy is a mechanism for the degradation of cytoplasmic components within the lysosomes that is conserved in animal species ranging from insects to worms and to mammals (Klionsky et al., 2003; Chang and Neufeld, 2010; Ravikumar et al., 2010; Tian et al., 2010; Russell et al., 2014; Bouché et al., 2016). At least 30 autophagy-related (Atg) proteins have been identified in yeast and mammals (Nakatogawa et al., 2009). The autophagy process starts with the encapsulation of the targeted molecules or organelles into a double-membrane structure called autophagosome (Klionsky et al., 2003). The interaction between Beclin 1 (homolog of the Vps30/Atg6) with the lipid kinase Vps34 (or PI3kC3, Phosphatydil-Inositol-3-kinase of class III) is fundamental in triggering the expansion and progression of the pre-autophagosomal structure that eventually leads to the formation of the autophagosome (Wirth et al., 2013). During this process, the microtubule-associated protein light chain 3 (MAP-LC3, the mammalian homolog of yeast atg8) is conjugated with phosphatidylethanolamine (to form PE-LC3 or LC3 II) and post-translationally inserted into the bilayer of the inner and outer membranes of the autophagosome (Kabeya et al., 2004). While in expansion, the pre-autophagosomal structure entraps the autophagy substrate that is tagged by p62/SQSTM1 (or equivalent) protein, which interacts with LC3II (Shvets et al., 2011). The autophagosome eventually fuses with several endosomes and lysosomes to form the autolysosome, wherein the autophagy substrate will be degraded along with the inner membrane of the autophagosome by lysosomal acid hydrolases (Glick et al., 2010; Barth et al., 2011; Klionsky et al., 2014). Basal autophagy contributes to the macromolecular turnover and to the maintenance of cellular homeostasis by degrading the aged and excessive cytoplasmic material and by replenishing the substrates needed for macromolecular synthesis (Russell et al., 2014; Kawabata and Yoshimori, 2016; Zaffagnini and Martens, 2016). In case of lack of nutrients (starvation), autophagy is triggered to sacrifice the redundant cytoplasmic material in order to recover the substrates to be recycled for the synthesis of vital cellular components (Glick et al., 2010; Tan and Miyamoto, 2016; Zaffagnini and Martens, 2016). The kinase mTOR (mammalian target of Rapamycin) integrates the information on the availability of nutrients and the need of energy, and controls both the macromolecular synthesis and the autophagy process (Dibble and Manning, 2013). Growth factors and amino acids keep mTOR active, thus promoting the synthetic processes and inhibiting autophagy. In contrast, the absence of amino acids reliefs the mTOR brake on autophagy, so that degradation of unused self-components is triggered in order to recruit the essential substrates needed for the production of energy and vital proteins and membranes (Settembre et al., 2013).

Therefore, autophagy is fundamental in preserving cell viability under nutrient shortage circumstances. For instance, shortly after delivery the neonate faces severe starvation until nutrients are supplied with milk, and during this period autophagy is triggered to maintain cell and tissue viability and to prevent perinatal death (Kuma et al., 2004). While during embryogenesis the level of autophagy remains low, it promptly rises in various tissues after birth and it is maintained at high levels for 3–12 h before returning to basal levels within 1–2 days (Kuma et al., 2004). Autophagy-defective mices appear normal at birth, yet they show reduced amino acid concentrations in plasma and tissues, and die within 1 day of delivery (Kuma et al., 2004).

Our knowledge on the effect of starvation on the reproductive organs of invertebrate species is very limited. It was reported that starvation stimulated autophagy in both germ cells and follicular cells in *Drosophila* ovaries (Barth et al., 2011). The homolog of LC3-II protein and of several other Atg proteins were significantly upregulated in germ cells following starvation. Recently, through gene mining and bioinformatics analyses of the transcriptomes, we have identified the presence of key autophagy genes in several organs, including the ovary, of the giant freshwater prawn *Macrobachium rosenbergii* (Suwansa-Ard et al., 2016).

*M. rosenbergii* is an important food source for Asian countries and the world. Nowadays, the aquaculture of this crustacean still faces many problems, including diseases and stress during captivity that result in lower fecundity and reproduction. There have been several attempts to increase the reproduction of this prawn by inducing gonad maturation, reducing the gonad development period and spawning using special formula feed (Cavalli et al., 2001; Takács-Vellai et al., 2005; Ribeiro et al., 2012) or through hormone injections (Tinikul et al., 2009; Sumpownon et al., 2015; Thongbuakaew et al., 2016a) or by eye-stalk ablation (Okumura and Aida, 2001).

A brief period of starvation has been shown to modify the lipid and protein contents in the ovary of the prawn *P. monodon* (Kawabata and Yoshimori, 2016) and to stimulate the oogenesis in drosophila (Chang and Neufeld, 2010). Whether, autophagy is stimulated in the gonads of the starved prawns and whether it associates with gonad maturation have not yet been investigated. Here, we have addressed these issues in the female *M. rosenbergii*. We found that a short period (up to 8 days) of starvation promoted ovarian maturation, yet when it was prolonged (at day 12) the ovarian maturation subsided and the prawns eventually died. The expression of autophagy markers concomitantly increased during starvation-induced ovarian maturion, reaching the highest level in late oocytes, implicating that autophagy and ovarian maturation were temporally correlated in starved prawn. We also showed that vitellin co-localized in autolysosome of mature oocytes of starved prawns. The present findings may pave the way for new strategies to stimulate oogenesis and reproduction of prawns in captivity.

## Materials and methods

### Animals

Adult females *M. rosenbergii* bought from a local commercial farm in Ayutthaya province, Thailand, were separated into fed and starved groups, each with 24 prawns. Six prawns from each group were randomly selected, anesthetized on ice water and sacrificed at days 1, 4, 8, and 12 (i.e., at 4 day intervals). The ovaries were dissected out to assess the gonado-somatic index (GSI). The GSI-values were calculated using the formula [ovarian weight (g)*/*body weight (g)] × 100. The ovaries were partly prepared for histological examination, and partly frozen in liquid nitrogen and kept at −80°C for Western blot analysis.

### Tissue preparation for histological examinations

Small pieces of the ovaries from each group were immediately immersed in a cold 0.4% paraformaldehyde fixative for 24 h. The specimens were then dehydrated in increasing concentrations of ethanol, cleared in xylene and infiltrated with paraffin, using an automated tissue processor. The paraffin-embedded tissue blocks were cut at 5 μm thickness using a Leica rotary microtome, placed onto silane-coated slides and stained with hematoxylin and eosin, or treated for immunofluorescence.

### Hematoxylin and eosin staining

Tissue sections were deparaffinized by xylene and rehydrated in a descending concentrations of ethanol, then they were stained with Harris's hematoxylin and eosin and mounted in a mounting medium. The ovarian stages, oocyte diameter and number of oocytes were evaluated by light microscopic observations. At least fifty randomly selected oocytes at each stage in three distinct ovarian sections from each prawn were visualized and photographed under a Nikon E600 light microscope, and images were recorded with a Nikon DXM 1200E digital camera. Each data set was expressed as a mean ± S.E.

### Estimation of ovarian cell proliferation by Ki67 staining

The ovarian sections were sequentially deparaffined and rehydrated in xylene and decreasing concentrations of ethanol, washed with 0.1 M phosphate buffered saline (PBS), pH 7.4, and permeabilized with 0.4% Triton-X 100 pH 7.4 (PBST). Subsequently, the epitopes of Ki67 protein were exposed with a warm 0.01 M Citric acid for 30 min, free aldehyde groups were blocked with 1% glycine in 0.1 M PBS for 30 min at room temperature, and then non-specific bindings were blocked with a blocking serum (10% fetal bovine serum in 0.1 M PBS) for 2 h at 4°C. The sections were incubated overnight at 4°C with rabbit anti-human Ki67 (Abcam) at a dilution of 1:200 in 5% blocking serum and subsequently incubated with goat anti- rabbit IgG-HRP as the secondary antibody (Invitrogen) at a dilution of 1:5,000 in 5% blocking serum for 2 h at room temperature. Finally, the color was developed with NBT/BCIP stock solution kit (Roche), then the reaction was blocked with 10 mM Tris–HCl, 1 mM EDTA; pH 8.1, for 10 min. The sections were mounted with glycerol buffer, and then viewed and photographed under a Nikon E600 light microscope. Five non-consecutive sections were taken from each ovary of 5 animals per group (*n* = 5) and examined at 40 × magnification. Data were expressed as numbers of dividing cells per mm^2^. The negative controls were performed by omitting the primary antibody.

### Immunofluorescence detection of atg proteins in the ovaries

The primary antibodies used to detect the autophagy markers in prawns were raised against the human protein homologs. In our previous study (Suwansa-Ard et al., 2016), we found that: 1. *M. rosenbergii* Beclin1 and the human ortholog *Homo sapiens* Beclin1 display about 60% similarity, share similar 3D conformation and show conserved aminoacid sequence in the functional domains for specific interactions with regulatory proteins (e.g., BCL-2, UVRAG, ecc); 2. the *M. rosenbergii* MAP1LC3 and human MAP1LC3B (HsaMAP1LC3B) share 72% similarity, and their structural superimposition indicated a similar secondary structure, including at the binding sites for Atg7 and tubulin; and 3. *M. rosenbergii* Lamp-1 mature protein contains a Lamp domain (position 40–324; Pfam accession number: PF01299), and the canonical transmembrane domain of the epidermal growth factor receptor (TM-EGFR) as in the human homolog. Also, the sequences of *M. rosenbergii* and human ATG proteins at the regions used for producing anti-Beclin1, anti-LC3, and anti-Lamp-1 antibodies shared 58.82, 42.86, and 23.37% identity. Accordingly, in that study we validated the cross-reactivity of these anti-human antibodies toward the corresponding *M. rosenbergii* Atg proteins (Suwansa-Ard et al., 2016). The specificities of the antibodies against LC3, Lamp1, and Beclin1 were tested by the manufacturer using standard immunohistochemical methods. Additionally, when the primary antibodies were omitted in our control sections no staining was detected, confirming their specific immunoreactivity toward the prawns Atg proteins. After the ovarian sections were deparaffined and rehydrated, free aldehyde groups were blocked with 1% glycine in 0.1 M PBS, and non-specific bindings were blocked with a blocking serum (10% fetal bovine serum in 0.1 M PBS) for 2 h at 4°C. They were then incubated overnight at 4°C with rabbit anti-microtubule-associated protein 1 light chain 3 (LC3) (Sigma-Aldrich, St Luois, US; L7347) diluted at 1:500 and/or monoclonal mouse anti-Lamp1 (BD Biosciences, 555798) diluted at 1:500 or polyclonal goat anti- Beclin 1 (Santa Cruz, sc-10086) diluted at 1:500, all in 5% blocking serum overnight at 4°C. After washing with PBS, the tissues were incubated for 2 h with secondary antibodies at room temperature in secondary antibodies: goat anti-rabbit IgG-FITC (Southern Biotech, Birmingham, US), goat anti-mouse IgG-TRITC (Southern Biotech) or goat anti-mouse IgG-FITC (Southern Biotech), or rabbit anti-goat IgG-FITC (Southern Biotech) at a dilution of 1:500 in 5% blocking solution. To determine the lysosomal localization of vitellin, the ovarian sections were incubated overnight at 4°C with monoclonal mouse anti-Lamp1 (BD Biosciences, 555798; diluted at 1:500 in 5% blocking serum) and with polyclonal rabbit anti-vitellin serum (at a dilution of 1:2,000 in 5% blocking serum). The latter was prepared in our laboratory as reported earlier (Soonklang et al., 2012). After washing with PBS, the tissues were incubated for 2 h at room temperature with the secondary antibodies goat anti-mouse IgG-FITC (Southern Biotech) and goat anti-rabbit IgG-TRITC (Southern Biotech) at a dilution of 1:500 in 5% blocking solution.

The tissues were washed three times with PBST and mounted in anti- fading mounting medium containing DAPI (Santa Cruz Biotechnology). Finally, the specimens were viewed and photographed under an Olympus FV1000 confocal laser scanning microscope (Olympus America, Center Valley, PA). Each tissue was scanned sequentially for each fluorophore to obtain separate images for each label and a merged image of the two or three channels was obtained for each optical section. The images were produced using subsets of the z-stacks. The stacking was performed using 3–5 images, each with the thickness of 1–2 micrometers. The staining intensity was scaled into three levels (weak, moderate, and strong) by comparison of multiple images of control and treated section, as independently assessed by two qualified investigators.

### Transmission electron microscopy

Small pieces of the ovaries were fixed in 2.5% glutaraldehyde and 2% paraformaldehyde in 0.1 M PBS at 4°C overnight and posted-fixed in 1% osmium tetroxide (OsO4) in 0.1 M PBS for 2 h at 4°C. Subsequently, they were washed with cold distilled water, dehydrated through increasing concentrations of ethanol (30, 50, 70, 80, 90, 95, and 100%), and infiltrated twice with propylene oxide for 30 min each. Samples were then placed in mixtures of propylene oxide and Araldite 502 resin at the ratios 2:1 for 30 min, 1:1 for 30 min, and 1:2 overnight. Samples were finally embedded in pure Aradite 502 resin and polymerized at 45 and 60°C, for 2 days each. For semithin sections, the specimens were cut at 600 nm thickness using a Sorvall MT-2 ultramicrotome, stained with 1% methylene blue and observed under a Nikon E600 light microscope and images recorded with a Nikon DXM 1200E digital camera. For ultrathin sections, the specimens were cut at 90 nm thickness, mounted on copper grids, and stained with lead citrate and uranyl acetate. Finally, the specimens were observed and photographed under a FEI-TECNAI 20 TWIN transmission electron microscope operating at 75 kV.

### Western blot analysis of LC3 and vitellogenin

The Western blotting of Atg protein markers in several organs in crustacean was performed following the protocol described previously (Suwansa-Ard et al., 2016). Briefly, frozen ovarian tissues were homogenized and sonicated in a lysis buffer (0.2% C_24_H_39_NaO_4_, 1 mM Na_3_VO_4_, and 50 mM NaF) and centrifuged at 10,000 × g to obtain tissue extracts. Supernatants were collected for measuring protein concentrations with Bradford reagent (Sigma-Aldrich). Subsequently, 30 μg of each protein extract was separated on 15% SDS-PAGE gel, and then transferred to a PVDF membrane (Millipore Corporation). The membranes were incubated in blocking buffer (5% non-fat dry milk in 0.2% Tween 20 in 0.1 M PBS, pH 7.4) for 2 h at room temperature to block non-specific bindings and then incubated overnight at 4°C with the following primary antibodies: rabbit anti-human microtubule-associated protein 1 light chain 3 (LC3) (Sigma-Aldrich, L7347) diluted at 1:500 or rabbit anti-vitellin at a dilution of 1:2,000 (Soonklang et al., 2012), and then they were incubated for 2 h at room temperature in peroxidase-conjugated goat anti-rabbit IgG (Southern Biotech) at dilutions of 1:5,000 and 12,000 for LC3 and vitellin, respectively. For comparison, tubulin protein in the extracts was stained with mouse anti- tubulin (Sigma-Aldrich) as the primary antibody and peroxidase-conjugated rabbit anti-mouse IgG at a dilution 1:10,000 as the secondary antibody. Immunoreactive bands were detected using an enhanced chemiluminescent substrate (Thermo Scientific) and exposed to Amersham Hyperfilm ECL (GE Healthcare Life Sciences).

### Statistical analysis

The data were presented as mean ± S.E. Statistical differences between two groups were analyzed by *t*-test, whereas comparisons between three or more groups were determined by one- way ANOVA. A probability value <0.05 (*p* < 0.05) indicated a significant difference (PRISM software version 3.03; GraphPad).

## Results

### Effects of starvation on gonado-somatic index and ovarian size

**Figure 1:**
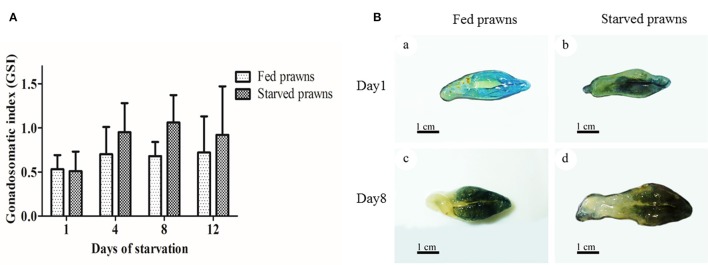
**Effect of starvation on gonado-somatic index (GSI) and ovarian size. (A)** Mean gonadosomatic index (GSI) of the starved prawns determined at days 1, 4, 8, and 12 compared to that of the fed prawns. **(B)** Gross morphology and sizes of the ovaries from fed and starved prawns at days1 and 8. *Bar* 1 cm **(a–d)** (*n* = 6).

A macroscopic examination revealed obvious differences in the color and size of the ovaries from fed and starved prawns (Figure 1). In the latter, the ovary appeared larger. The gonado-somatic index (GSI), an objective measurement of the weight of the gonad with respect to the body weight of the whole organism, confirmed the progressive increase of the size of the ovaries along with the time of starvation (Figure 1A). While the GSI remained unvaried in control prawns during the 12 days of observation, the GSI increased between 1 and 4 days of starvation, and further increased after 8 days of starvation, then it slightly decreased by day 12. At any time point considered, except for day 1, the GSI was higher in the starved prawns as compared to the control counterpart. At day 1, the ovaries of both starved and fed prawns exhibited similar small and pale yellow doughnut-shaped masses (Figures 1Ba,b). In contrast, at day 8 the ovaries of starved prawns increased in size and appeared as dark green doughnut-shaped masses, compared to those of fed prawns (Figures 1Bc,d).

### Effects of starvation on histology and maturation of the oocytes

**Figure 2:**
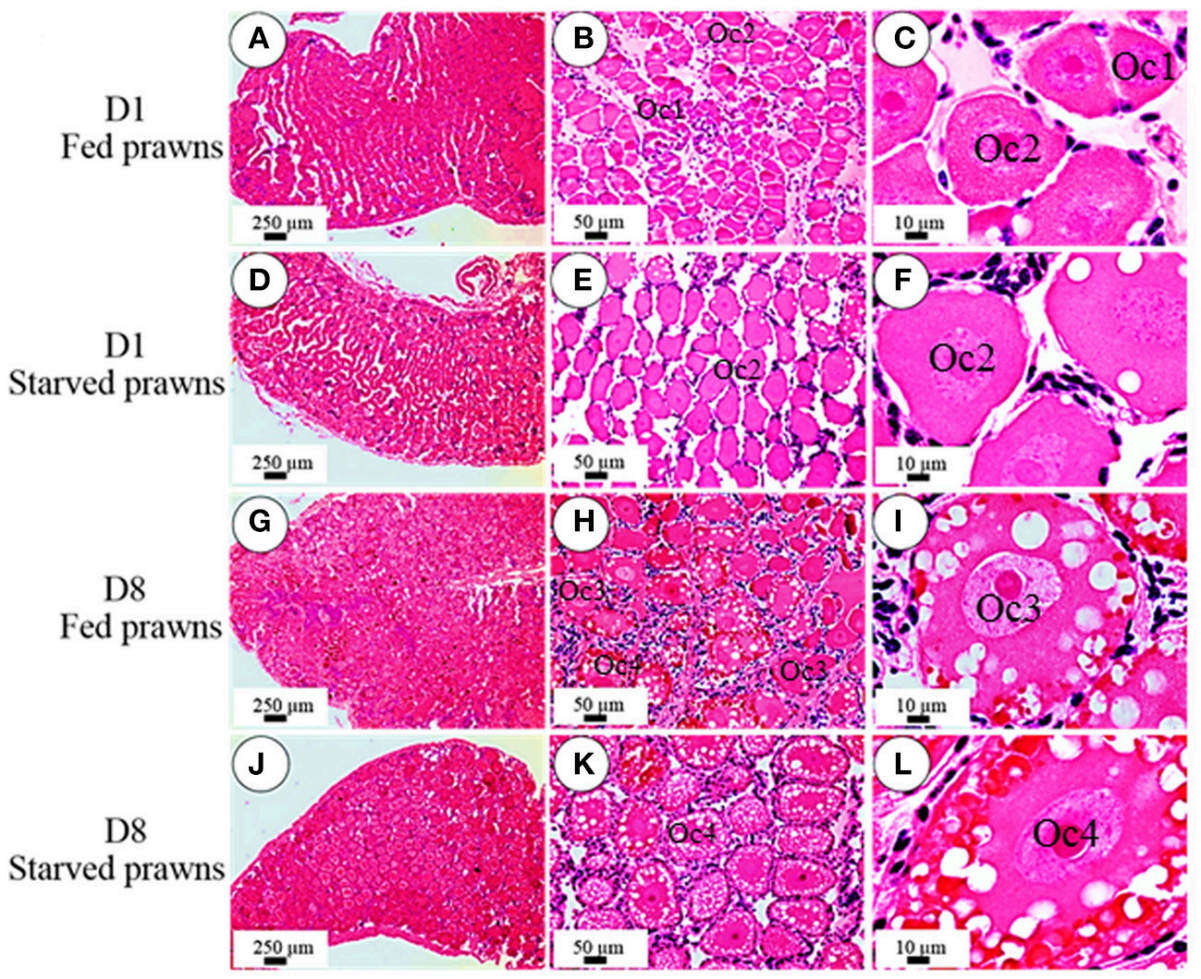
**Effect of starvation on ovarian histology and maturation of the oocytes**. Light micrographs of hematoxylin and eosin- stained ovarian sections showing various stages of oocytes in the ovaries of fed prawns at day1 **(A–C)**, starved prawns at day1 **(D–F)**, fed prawns at day 8 **(G–I)** and starved prawn at day 8 **(J–L)**. *Bar* 250 μm **(A,D,G,J)**, 50 μm **(B,E,H,K)**, 10 μm **(C,F,I,L)** (*n* = 6).

**Figure 3:**
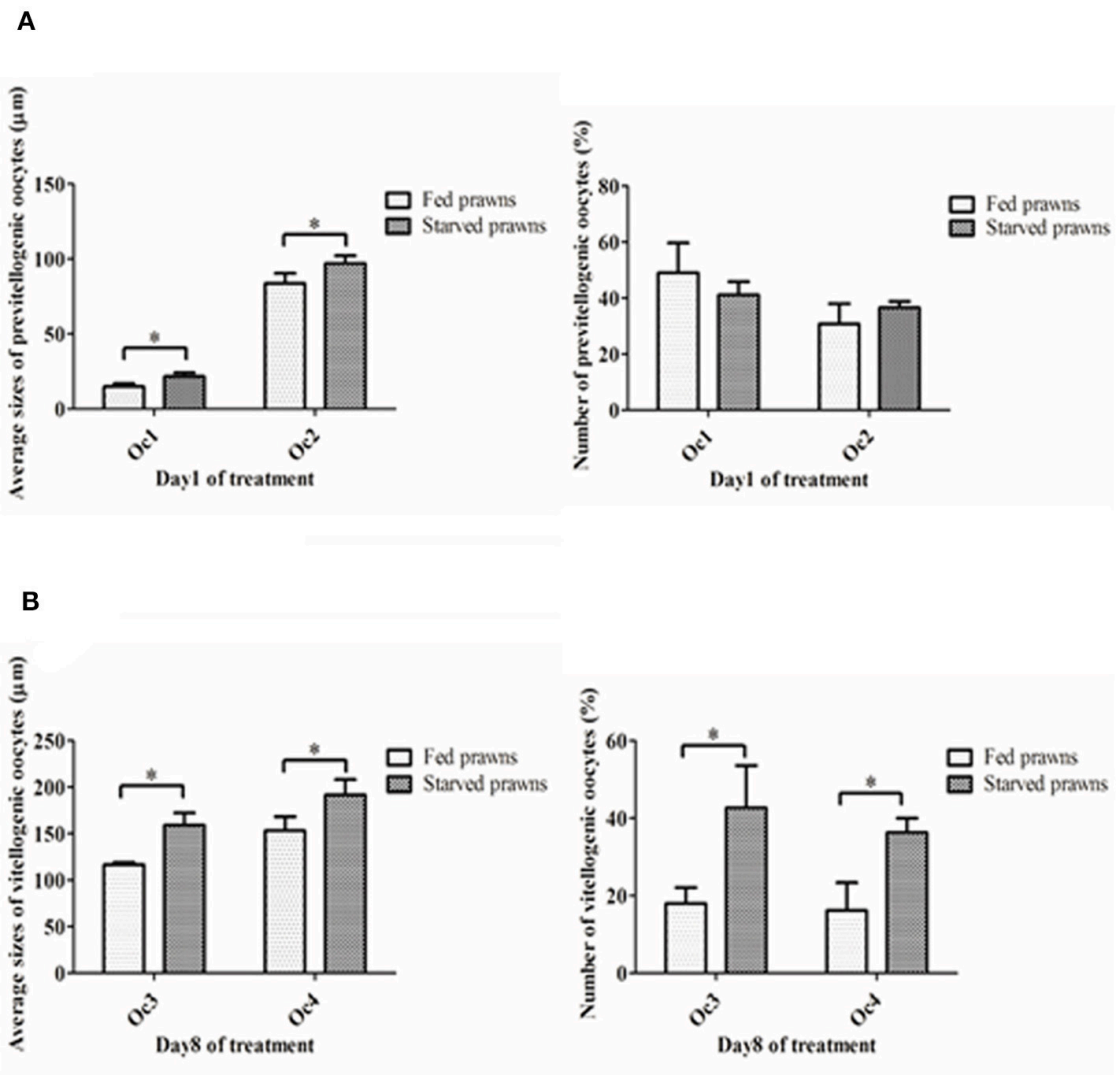
**(A)** Mean oocyte diameters and numbers of pre-vitellogenic oocytes (Oc1, Oc2) of the starved prawns at day1 compared to that of the fed prawns. **(B)** Mean oocyte diameters and numbers of vitellogenic oocytes (Oc3 and Oc4) of the starved prawns at day 8 compared to that of the fed prawns. Asterisks indicate significant differences compared to the fed group with *p* < 0.05. (*n* = 50 randomly selected oocytes per ovary).

According to the criteria proposed in our previous publications (Meeratana and Sobhon, 2007; Tinikul et al., 2008; Soonklang et al., 2012), the ovarian cycle of *M. rosenbergii* is classified into 5 stages based on coloration, size and composition of oocytes in the ovary. On histologic examination, the ovaries from either starved and fed prawns at day 1 were in proliferative phase (stage II ovary) and contained mostly proliferating germ cells (oogonia, Og) and previtellogenic oocytes (Oc1 and Oc2) (Figures 2A–F), while the ovarian histology of both groups at day 8 showed that the ovaries were in stage IV(mature phase) and contained mainly vitellogenic oocytes (Oc3 and Oc4). However, the ovaries of starved prawns contained more Oc4 than Oc3 and these cells appeared larger in size (Figures 2G–L). The average sizes of the previtellogenic oocytes were significantly larger in the starvation group compared to the fed group (Figure 3A) whereas the average sizes and the numbers of these vitellogenic oocytes were significantly increased in starved prawns compared to those of fed prawns (Figure 3B).

### Effect of starvation on ovarian cell proliferation

**Figure 4:**
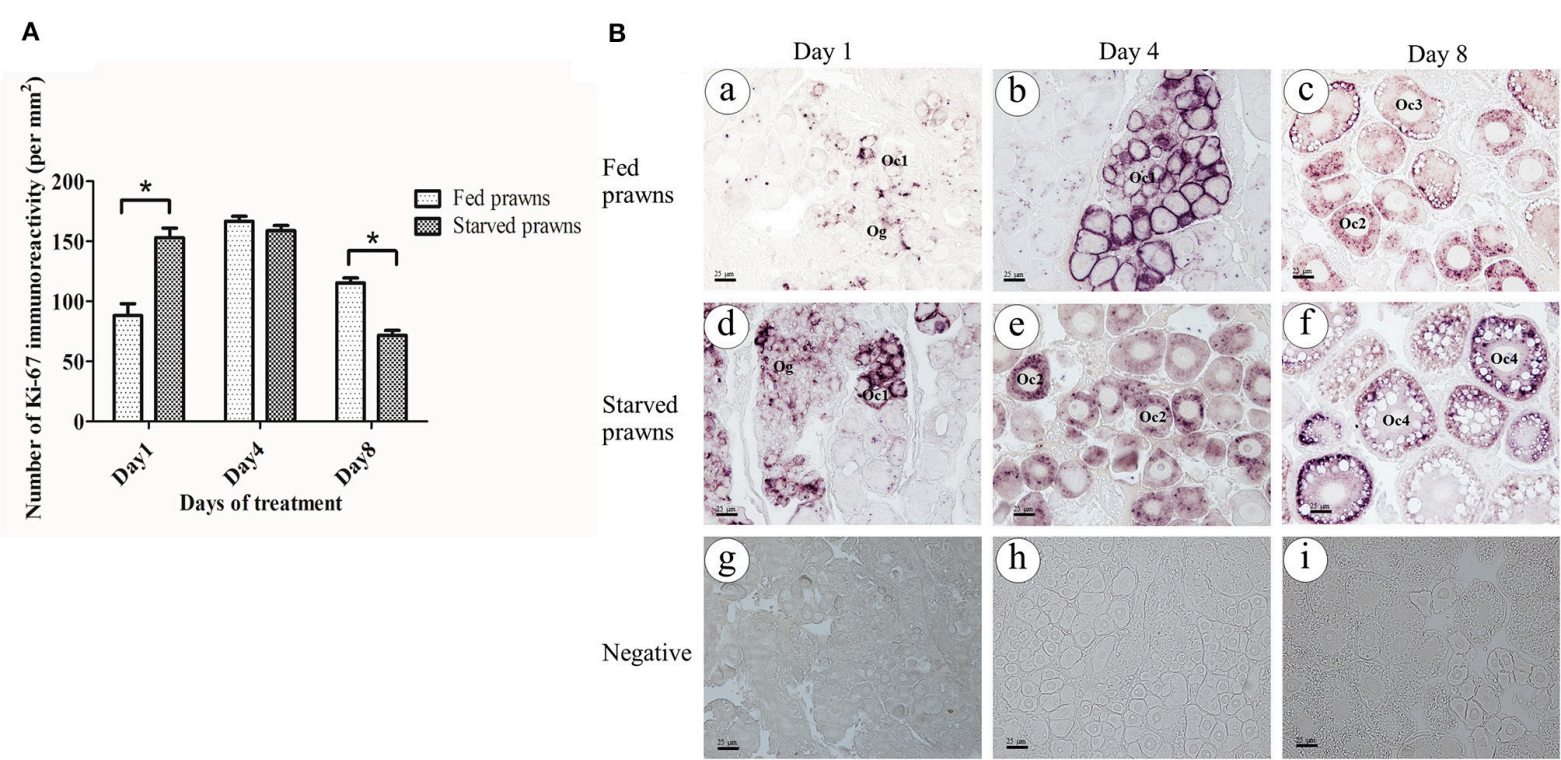
**Effect of starvation on ovarian cell proliferation**. Immunoreactivity of Ki67 (number of positive cells/area) in ovaries of starved prawns significantly increased at day 1 and thereafter decreased at day 8 compared to fed prawns **(A)**. At day 1, the number of positively stained oogonia and Oc1 in the fed prawns'ovaries **(Ba)** were lower than in starved prawns **(Bd)**. At day 4, fed prawns'ovaries **(Bb)** showed immunoreactive oogonia and Oc1, whereas starved prawns'ovaries **(Be)** exhibited immunoreactive Oc1 and Oc2. By day 8, fed prawns'ovaries showed immunoreactive Oc3 **(Bc)**, whereas starved prawns'ovaries showed more intense immunoreactive Oc4 **(Bf)**. Negative control in all groups did not show Ki67 immunoreactivity **(Bg–Bi)**. *Bar* 25 μm (*n* = 5). Asterisks indicate significant differences compared to fed group with *p* < 0.05.

The presence of immunoreactivity of Ki67, which reflects the cell proliferation, was investigated in the ovaries of both fed and starved prawns (Figures 4A,B). When compared the density of Ki67 positive cells (number of immunopositive cells/section area) the ovaries of the two groups showed a different kinetics: cell proliferation was stimulated by starvation since day 1 and remained stable for a few days, then it declined to the lowest level at day 8. On the contrary, in fed prawns cell proliferation reached the peaked at day 4, and slightly declined at day 8, though remaining higher than in starved oocytes. Focusing on cell types, at day 1, the ovaries of fed prawns (Figure 4Ba) showed fewer Ki67 positive oogonia and Oc1 compared to the starved prawns (Figure 4Bd). By day 4, the ovaries of fed prawns showed Ki67 positivity in the Oc1 (Figure 4Bb), whereas starved prawn showed Ki67 positivity in both Oc1 and Oc2 cells (Figure 4Be). At day 8, fed prawn showed Ki67 positivity mostly in Oc3 (Figure 4Bc), while starved prawn showed higher Ki67 positivity in Oc4 (Figure 4Bf). Negative controls were done by omitting the primary antibody in consecutive sections (Figures 4Bg–i), which confirmed the specificity of the immunoreactivity.

### Immunofluorescence localization of autophagy markers in oocytes

**Figure 5:**
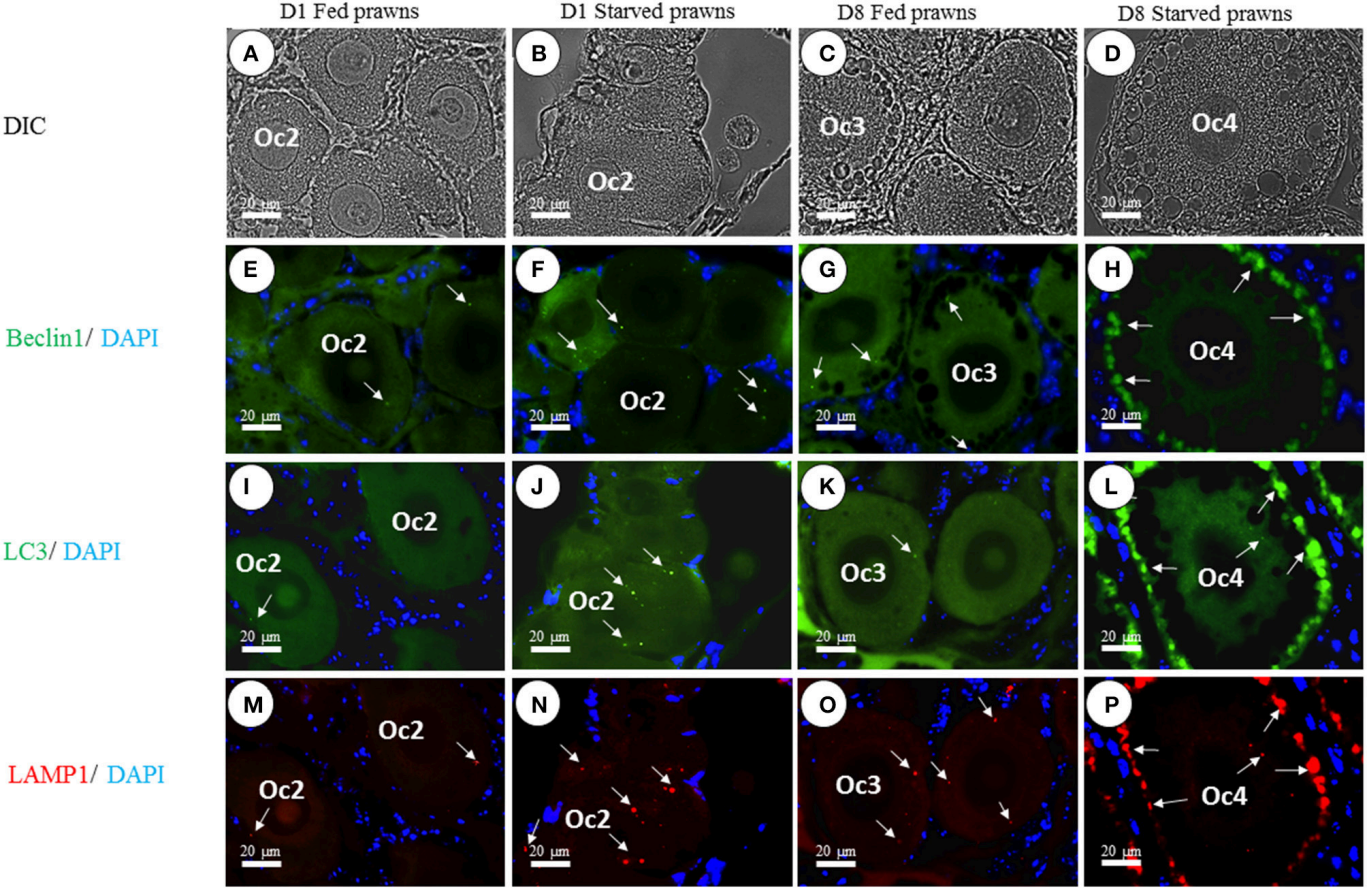
**Effect of starvation on autophagy in the oocytes**. The immunoreactivity of autophagic-related proteins (ARP) in various stages of oocytes from Oc1 to Oc4 at days 1 and 8 was shown in DIC **(A–D)**. Lower panels showed immunofluorescence detection of Beclin1 (arrows in **E–H**), LC3 (arrows in **I–L**), and LAMP1 (arrows in **M–P**). It was evident that the immunoreactivities of ARP were more intense in the ovaries of starved prawns, especially in late stage oocytes. *Bar* 20 μm (*n* = 5).

Previous data showed that starvation could influence cell proliferation and maturation in the ovary. Starvation is a well-known trigger of autophagy, a cellular stress response that plays a role in the control of cell proliferation and differentiation. In the search for a possible link between autophagy and oocyte maturation, we examined the ovaries for the presence of autophagy markers Beclin1, LC3, and Lamp1. Beclin1 is an essential activator of Vps34 for the initiation of the autophagy process (McKnight and Zhenyu, 2013), LC3 (isoform II) is a protein that is post-translationally inserted into the nascent autophagosomal membranes (Slobodkin and Elazar, 2013), and Lamp-1 is an integral membrane protein of endosomes and lysosomes that plays a role in the fusion of these organelles with autophagosomes (Eskelinen, 2006). Of the three, LC3 is considered a reliable hallmark of autophagosome formation (Klionsky et al., 2016). This protein is bound to tubulin when is in the “non-autophagy active” MAP-LC3 form. Once autophagy has been triggered, this protein is processed by ATG4 and detaches from tubulin and the Glycine at its C-terminus is exposed to generate a soluble LC3-I isoform. LC3-I is subsequently conjugated (by an ATG5-ATG7 complex) with Phosphatydil-Ethanolamin to generate LC3-PE (better known as LC3-II isoform), which is finally translocated into the lipid bylayer of the inner and outer autophagosomal membranes. When the LC3-positive autophagosome fuses with the Lamp1-positive endosomes and lysosomes, an LC3-Lamp1 double positive autolysosome forms. We have previously reported on the immunohistochemical localization of Atg protein markers during various ovarian stages in *M. rosenbergii* (Suwansa-Ard et al., 2016). Here, we compared the level of expression of Beclin1, LC3, and Lamp1 in Oc2 to Oc4 cells in the ovaries of both fed and starved prawns. The images in Figure 5 show that the positivity for these Atg markers is much higher in the oocytes of starved prawns. When immunoreactivity of the individual Atg proteins was compared, Beclin1 exhibited moderate intensity (Figures 5E–H), whereas LC3 (Figures 5I–L) and Lamp1 (Figures 5M–P) exhibited relatively higher intensity. Notably, the immunoreactivity of these three Atg proteins increased toward the peripheral cytoplasm of the Oc4, especially in the ovaries of starved prawns at day 8 (Figures 5H,L,P). In particular, LC3-Lamp1 double-positive autolysosomes accumulated at the extreme periphery of the cell, beneath the plasmamembrane. Interestingly, a strong positivity for these three autophagy markers was not detected in follicular cells of the starved ovaries.

### Ultrastructure of the ovaries confirms the presence of autophagic vacuoles in the oocytes of starved prawns

**Figure 6:**
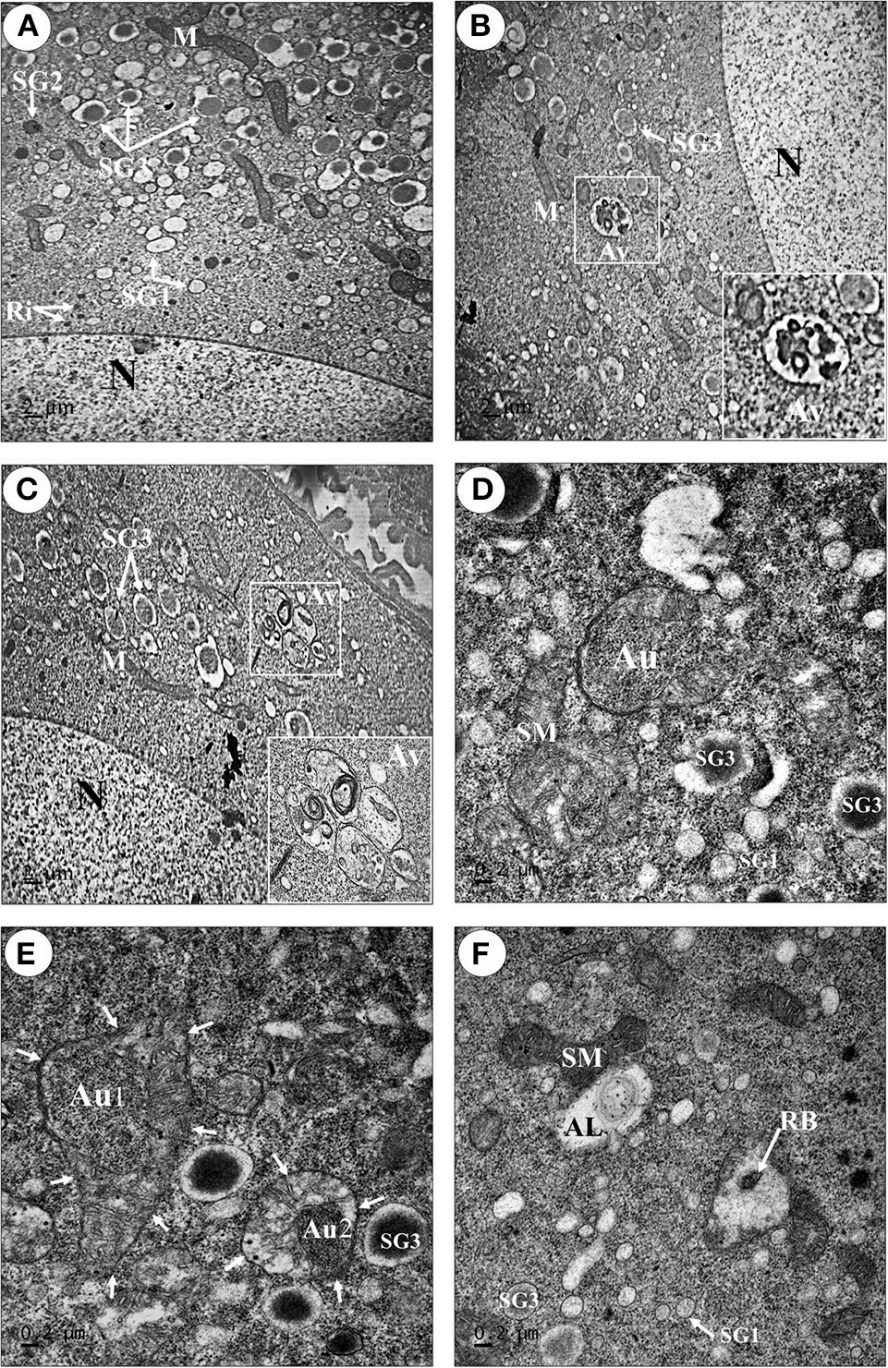
**Transmission electron micrographs showing an increase in number of autophagic vacuoles in the late stage oocytes of starved prawns**. An oocyte (Oc4) of fed prawn **(A)** showed euchromatic nucleus (N) and cytoplasmic organelles including mitochondria (M), ribosomes (Ri), and three types of secretory granule, whereas in addition to these organelles the oocytes of starved prawn **(B–F)** exhibited an increase in number of swollen mitochondria (SM), phagophore assembly site (PAS) which was suggestive of the early step in the formation of autophagosome (Au) (**D,E**; Au1), PAS eventually closed and engulfed the mitochondrion forming complete Au (**E**; Au2). Autolysosome (AL) and residual body (RB) were also observed in the cytoplasm of starve prawn. SG1, secretoty granule type1; SG2, secretory granule type2; SG3, secretoty granule type3. *Bar* 2 μm **(A–C)**, 0.2 μm **(D–F)** (*n* = 3).

Above data are suggestive of the ongoing autophagy process in the ovary. Electron microscopy was performed in order to gain more substantial proofs of the induction of autophagy during the oocyte maturation. The ultrastructure of oocytes at stage 4 (Oc4) of fed (Figure 6A) and starved prawns (Figures 6B–F) were compared. The Oc4 of both groups presented similar basic features, with the nucleus containing mainly euchromatin and the cytoplasm containing numerous organelles including free ribosomes at the perinuclear region, three types of secretory granules (SG1, SG2, and SG3), lipid droplets and several mitochondria (Figures 6A,B). Remarkably, a high number of autophagic vacuoles at different steps of maturation could be detected in the cytoplasm adjacent to the nucleus of Oc4 in the ovary from starved prawn (Figures 6B,C). Autophagosomes, typically circled by a double-membrane, were clearly detectable in these cells (Figure 6D). Swollen mitochondria and a mitochondrion within the autophagosome were clearly recognizable (Figure 6D). Various steps of the autophagy process as induced by starvation could be clearly observed. The mitochondria positioned in an area adjacent to a phagophore assembly site (PAS; Figure 6E) is suggestive of the early step in autophagosome formation. After an initial expansion, this structure eventually closed and engulfed the mitochondria (Figure 6E). At this point, two distinct membranes surround the autophagosome (Figure 6E). Numerous swollen mitochondria were also found within the autophagosomes in the oocytes of starved prawns (Figure 6F). When the autophagosome fuses with lysosomes, an autolysosome is formed, wherein the hydrolytic enzymes degrade the cytoplasm-derived materials along with the inner membrane of the autophagosome. Degraded products are transported back to the cytoplasm for recycling, while undigested products remain in the vesicle known as residual bodies (Figure 6F).

### Starvation promotes the production of vitellin proteins and lysosomal co-localization with vitellin in yolk granules

**Figure 7:**
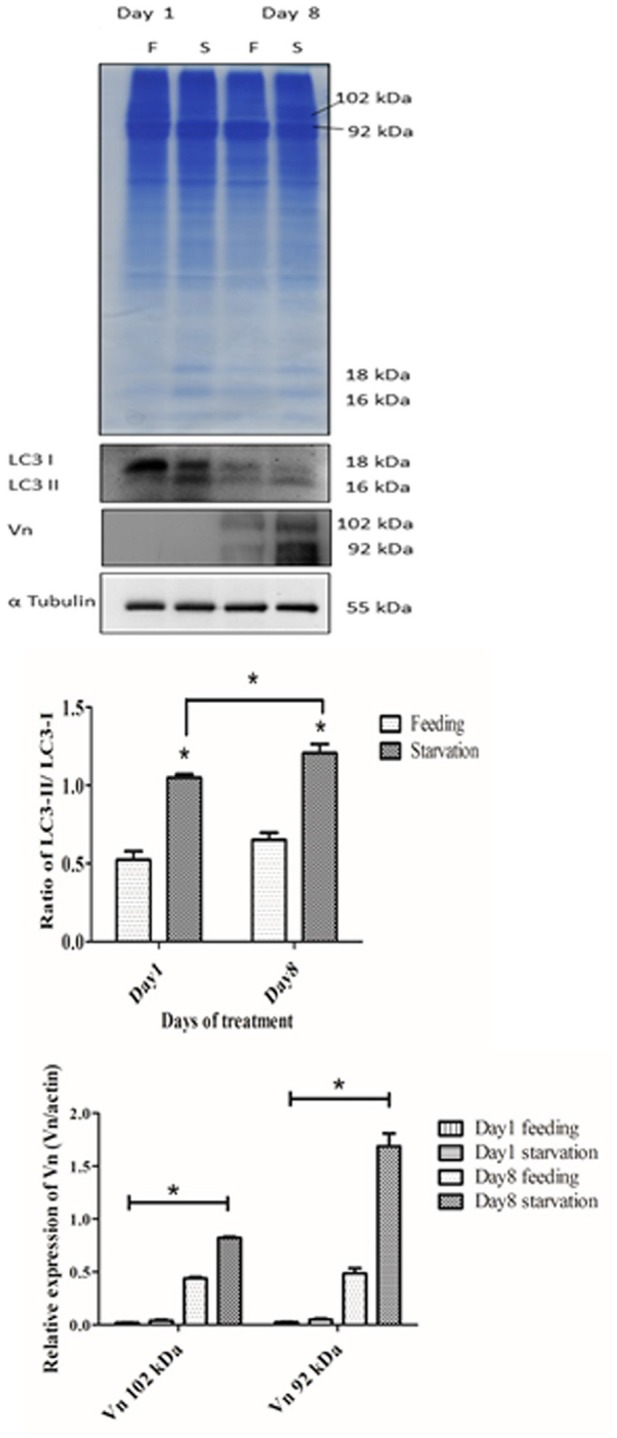
**Effect of starvation on the expressions of autophagic marker proteins and vitellin (Vn) in the ovaries**. Immunoblot showing the relative levels of LC3 I and II, Vn and β-tubulin in ovaries of fed and starved prawns. Both LC3-II and Vn showed significantly increased amounts in starved prawns'ovaries in comparison to fed prawns'ovaries, especially at day 8 (*n* = 5). Asterisks indicate significant differences with *p* < 0.05.

**Figure 8:**
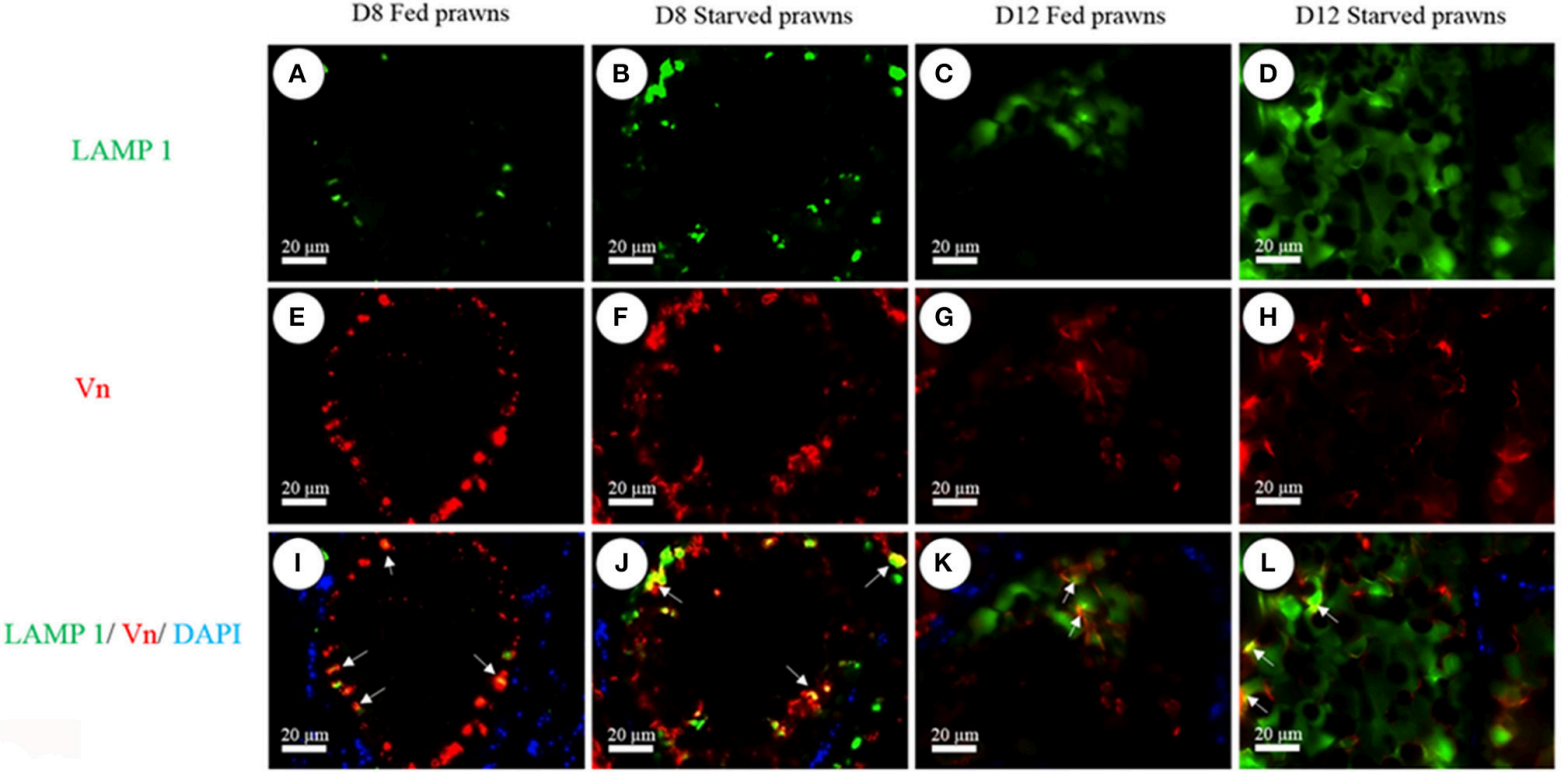
**Immunofluorescence detection of Lamp1 (A–D)** and Vn **(E–H)** in Oc4 of the ovaries. Nuclei were counterstained with DAPI. Note the colocalization of Lamp1 and Vn immunoreactivities in the merged images of Oc4 **(I–L)**. *Bar* 20 μm (*n* = 5).

As an additional proof of autophagy induction by starvation, we checked by Western blotting the generation of the lipidated form of LC3 (i.e., isoform II), which is an hallmark of autophagosomes. Homogenates from ovaries of both starved and fed prawn presented two LC3 immunoreactive bands, the upper one representing the cytosolic isoform LC3-I and the lower one representing the autophagosome membrane-associated LC3-II isoform (Figure 7). It was evidently clear that, in the starved ovaries, the amount of LC3-II relative to LC3-I was increased at day 1 and day 8, compared to the LC3-II/LC3-I ratio in the fed ovaries (Figure 7). In addition, at day 8 the amounts of ovarian vitellin (Vn) at both 92 and 102 kDa were significantly higher in starved than in fed prawns (Figure 7). In the search for a possible link between the two phenomena (production of vitellogenin subproducts and autophagy), we performed a double-staining of Vn proteins and the autolysosome marker Lamp1. Immunofluorescence at days 8 and 12 clearly showed the co-localization of Lamp1 and Vn in the cytoplasm of Oc4 of starved prawns, which implies a functional relationship between autophagy and Vn metabolism during oocyte maturation (Figures 8A–L).

## Discussion

Autophagy is an evolutionarily conserved mechanism for the degradation of cytoplasmic components within the lysosomes that allows the nutrient recycling during starvation (Kuma et al., 2004; Settembre et al., 2013). The aim of the present study was to investigate the effects of starvation on the modulation of autophagy in the ovaries of female *M. rosenbergii* and see if there was a functional involvement of autophagy in the oocyte maturation process. The major findings of this study were: (1) starved prawns showed a shorter time of ovarian maturation and an increase in the dimension and number of mature oocytes; (2) the expression of autophagy markers was upregulated in late oocytes (Oc3, Oc4) and in the follicular cells of starved prawns; the presence of autophagy vacuoles in starved oocytes was confirmed by ultrastructural studies; (3) Vn proteins was co-localized with Lamp1, a marker of autolysosomes, in the yolk granules of Oc4.

The ovarian cycle of *M. rosenbergii* is divided into five stages based on coloration, size and composition of oocytes in the ovaries (Okumura and Aida, 2001; Meeratana and Sobhon, 2007; Soonklang et al., 2012). Stage I is a spent stage, when the ovary contains mostly connective tissue scaffold and appears as a translucent organ located dorsal to the gut and hepatopancreas. Oogonia (Og), primary oocytes, small numbers of early and late previtellogenic oocytes (Oc1 and Oc2) are found in each ovarian pouch. Stage II is a proliferative stage, when the ovary shows yellow doughnut-shaped mass overlying the hepatopancreas. The ovary contains Og, Oc1, Oc2 and early vitellogenic oocyte (Oc3), with Oc2 and Oc3 being predominant cells. Stage III is a pre-mature stage, when the ovary shows dark green color with significant increase in size, and contains Og, Oc1, and mostly early vitellogenic oocytes (Oc3). Stage IV is the mature stage, when the ovary is increased in size covering the hepatopancreas and extends to the first abdominal segment. The ovary in this stage contains mostly mature oocytes (Oc4) and a few Og, while Oc1, Oc2, Oc3 are absent. Stage V is the spawn stage, when the Oc4 are released from the ovary, and groups of Og start to appear and proliferate at the central ovarian core. The ovary is surrounded by the thickened and folded ovarian capsule. We found that the ovaries undertook a rapid maturation process following starvation, as demonstrated by the typical changes in coloration and by histology, with a significant increase in the average size and numbers of vitellogenic oocytes (Oc3 and Oc4), compared to the ovary of fed prawns. Hence, we suggest that a short period of starvation could induce ovarian maturation and development of the vitellogenic oocytes.

Starvation is known to induce autophagy, resulting in the digestion and degradation of self-proteins and damaged organelles within the lysosome. The genes and proteins that control autophagy have been studied in a variety of organisms, including the yeast and several species of mammals and invertebrates. We have been the first in describing the genes and the proteins associated with the autophagy machinery in the giant prawn *M. rosenbergii* (Suwansa-Ard et al., 2016). In that study, we have validated the expression of Atg genes and proteins, including Beclin1 (BECN1), LC3 (MAP1LC3B), and Lamp1 in the oocytes (especially in the late stage) of the prawn (Suwansa-Ard et al., 2016). Interestingly, the association between autophagy and reproduction in the invertebrate was recently described by Barth et al. (2011), who suggested that the autophagy process induced by starvation could play a role in oogenesis in *D. melanogaster*. Recently, our group had identified estradiol and progesterone through LC-MS/MS analysis of the central nervous system, hepatopancreas and ovary of *M. rosenbergii* (Thongbuakaew et al., 2016b). In female prawn, progesterone stimulates ovarian maturation and spawning, whereas estradiol triggered yolk protein systhesis (Summavielle et al., 2003; Martins et al., 2007; Coccia et al., 2010). Moreover, gene mining analysis found estrogen- and progesterone- like receptors in the eyestalk, central nervous system, and ovary of *M. rosenbergii* (Thongbuakaew et al., 2016b). The expression of estrogen receptors in ovarian cells was increased in caloric restriction every other day in female mice (Sluczanowska-Glabowska et al., 2015). Caloric restriction increases the ratio of estrogen to androgen receptors expression in murine ovaries, with potential therapeutic implications (Sluczanowska-Glabowska et al., 2015). By analogy, we assume that nutrient limitation positively impacted on endocrine stimulation of the ovary in female prawns, and this likely would reflect on improved reproductive activity at the initial period. However, if starvation is prolonged the reproductive activity subsides.

Our study is the first to report the effects of starvation on autophagy and ovary maturation in a prawn. Immunofluorescence showed that autophagy markers, including Beclin1, LC3 and Lamp1, were upregulated in the ovaries of starved prawns, especially in the vitellogenic oocytes. Ultrastructural studies revealed the presence of a high number of autophagic vacuoles at different stages of maturation in the cytoplasm of late oocytes of starved prawns, strongly suggesting a functional link between the autophagy process and the concurrent maturation of the oocytes.

The key event in the oocyte development is the process of yolk synthesis or vitellogenesis (Tsukimura, 2001; Okuno et al., 2002; Nagaraju, 2011; Revathi et al., 2012; Reddy et al., 2013). Vitellogenesis in *M. rosenbergii* begins with the synthesis of vitellogenin in the hepatopancreas, then it is released into the hemolymph and cleaved into vitellin, which is taken up and incorporated into the yolk granules in the late oocytes, i.e., Oc3 and Oc4 (Okuno et al., 2002). Vn proteins show relative masses at 90 and 102 kDa, and the immunoreactivities of both proteins can be observed in the cytoplasm of vitellogenic oocytes (Oc3 and Oc4) (Soonklang et al., 2012). In this study, Vn was highly expressed in Oc3 and Oc4 in the ovaries of starved prawns, as compared to the fed prawns. Consistent with a possible functional link, in the starved ovary the autophagosomal marker LC3-II was highly expressed, and was localized in the vitellogenic oocytes together with Lamp1 and Beclin1. In addition, Vn co-localizated with Lamp1 in the oocytes of starved ovary. Whether the production of vitellogenin sub-products and autophagy are mechanistically correlated remain to be demonstrated through pharmacologic and genetic manipulations. Autophagy is a stress-response to starvation aimed at maintaining cell homeostasis and survival. We can hypothesize that by day 8 of starvation autophagy is upregulated beyond a critical point for cell survival, and that autophagy-mediated degradation of Vn is initiated in an attempt to preserve oocyte viability. Starvation-induced autophagy of the Vn granules could also serve to provide nutrients and substrates useful for the maturation of the oocyte. This interpretation is supported by similar findings reported in other experimental models. It was previously reported that vitellogenic secretion by the mosquito fat body was terminated by lysosomes. In this study, the mature Vn-containing secretory granules were shown to directly fuse with lysosomes (Raikhel, 1986). In a more recent study, the autophagy-incompetent female mosquitoes were unable to complete the reproductive cycle and exhibited retardation and abnormalities in egg maturation, while the autophagy competent wild type showed an intense lysosomal activity directed at the specific degradation of Vn (Bryant and Raikhel, 2011).

In conclusion, the present original findings provide novel insights on the functional involvement of starvation-induced autophagy in the ovarian maturation. A short period of starvation in female *M. rosenbergii* stimulated ovarian maturation and increased autophagic process in vitellogenic oocytes, likely promoting the utilization of yolk proteins during oocyte maturation. Yet, it must be stressed that prolonged autophagy may elicit negative effects, and in fact the ovaries of starved prawns at day 12 clearly showed shrinkage. We propose that a short period of starvation could be applied to induce ovarian maturation in the female broodstock, and this should be followed by re-feeding in order to sustain the ovarian maturation and oocyte production.

A better understanding of the mechanistic relationship between autophagy and ovary maturation could open to new strategies to induce oogenesis and thus improve the reproduction of prawns in captivity. Studies in our laboratories are ongoing to test this hypothesis and see whether it can be successful in aquaculture system.

## Ethics statement

This study was carried out in accordance with ethical standards.

## Author contributions

WK: Data acquisition, data analysis and interpretation, and manuscript preparation. CW: Data acquisition, manuscript preparation and final approval. AE, CS, MM, and MN: Data acquisition. RT and WS: Data analysis and interpretation. FM: Data analysis and interpretation. CI and PS: Conception and design of study and final approval.

### Conflict of interest statement

The authors declare that the research was conducted in the absence of any commercial or financial relationships that could be construed as a potential conflict of interest.
